# Understanding spatial heterogeneity of rocky reef demersal fish assemblages: Links to conservation plans in the Juan Fernández Archipelago Marine Protected Area in the Southeastern Pacific

**DOI:** 10.1371/journal.pone.0358459

**Published:** 2026-10-09

**Authors:** Braulio Tapia, Billy Ernst, Stéphane Gauthier, Cole C. Monnahan, Carolina Parada, Pablo Rivara, Noble Hendrix

**Affiliations:** 1 Programa de Magíster en Ciencias con mención en Pesquerías, Universidad de Concepción, Concepción, Chile; 2 Centro de Estudios Avanzados en Zonas Áridas (CEAZA), Coquimbo, Chile; 3 Departamento de Oceanografía, Universidad de Concepción, Barrio Universitario s/n, Concepción, Chile; 4 Millennium Nucleus Ecology and Sustainable Management of Oceanic Islands (ESMOI), Coquimbo, Chile; 5 Fisheries and Oceans Canada, Institute of Ocean Sciences, Sidney, British Columbia, Canada; 6 Department of Biology, University of Victoria, Victoria, British Columbia, Canada; 7 Resource Ecology and Fisheries Management Division, Alaska Fisheries Science Center, National Oceanic and Atmospheric Administration (NOAA), Seattle, Washington, United States of America; 8 Departamento de Geofísica, Universidad de Concepción, Barrio Universitario s/n, Concepción, Chile; 9 QEDA Consulting, LLC, Seattle, Washington, United States of America; The University of Auckland - City Campus: University of Auckland, NEW ZEALAND

## Abstract

Spatial heterogeneity linked to fish assemblages has been key to advancing conservation plans and assessing marine resources in remote ecosystems. The Juan Fernández Archipelago is a remote and highly heterogeneous geologic hotspot located on the Nazca plate (SE Pacific), comprising three islands, numerous seamounts and guyots, and a highly rugged coastal marine shelf. Hundreds of rocky reefs of various sizes characterize this insular coastal shelf, supporting diverse finfish populations of interest for the Robinson Crusoe Island artisanal fishing fleet. Two hydroacoustic surveys were conducted on the insular shelf of Robinson Crusoe and Santa Clara Islands. Reef distribution and morphometric attributes (length, height, depth strata, and geographic zones) were characterized across the shelf seascape. To identify traits that correlated with the presence and acoustic backscatter of demersal fish aggregations, a set of statistical models was implemented. Results indicate that reefs were present up to ~200 meters in depth around the islands, with the northern region hosting small and tall reefs, while the southern region had the fewest reefs but the largest. The findings indicated that 75% of the fish aggregations observed outside of reefs were within 280 m of them. Also, there was a higher probability of fish aggregations occurring in depths up to 150 m (~90%) given the presence of reefs, compared to the relatively flat seafloor (~20%). Reef–scale analysis showed that the longer and taller the reef, the higher the acoustic backscatter from fish. These findings highlight the ecological value of reefs for fish populations throughout the insular shelf seascape. In addition, this study highlights the usefulness of small–scale hydroacoustic surveys as a monitoring tool to assess the association between ocean–floor spatial heterogeneity and demersal fish assemblages, and the implementation of effective periodic surveys within the Juan Fernández Marine Protected Area.

## Introduction

The study of landscapes has made significant progress in terrestrial ecology, identifying critical aspects of habitat definition at different scales, an interplay among geological, environmental, and biological features [1–3]. Similarly, this interplay can be observed at seascapes capable of structuring populations and communities, such as intertidal habitats, seagrasses, seamounts, and reef seascapes [4–6]. In particular, insular shelves around oceanic islands often harbor numerous geographic features and oceanographic characteristics that create high spatial habitat heterogeneity, shaping the local distribution of populations and communities [7–9].

The rugged coastal shelf of the Juan Fernández Archipelago (JFA) off the Chilean coast (Fig 1) constitutes an ideal seascape for studying spatial ecological patterns underlying the marine environment. From volcanic matter deposits associated with geologic hotspots, and the subsequent cooling of the tectonic plate [8,11], a conglomerate of oceanic islands and guyots emerged, constituting the Juan Fernández Ridge. This seamount string is oriented perpendicular to the southwestern edge of the South American continent (SE Pacific, Fig 1B; [12]). JFA presents numerous reefs on its shelves, modulated by geomorphic processes, basaltic composition [13], and sedimentary deposits [11]. This region shelters an extensive reservoir of endemic fish that extends its distribution to the island shelf break (~200 m), forming dense assemblages in both the pelagic and benthic environments [14,15]. Thus, the JFA constitutes a vulnerable marine ecosystem and reservoir for numerous endemic species [16,17], which is embedded in a Multiple Use Coastal Marine Protected Area (AMCP–MU) entitled “Juan Fernández Sea” (Supreme Decree N°10 of 2018, Chile [18]).

**Fig 1:**
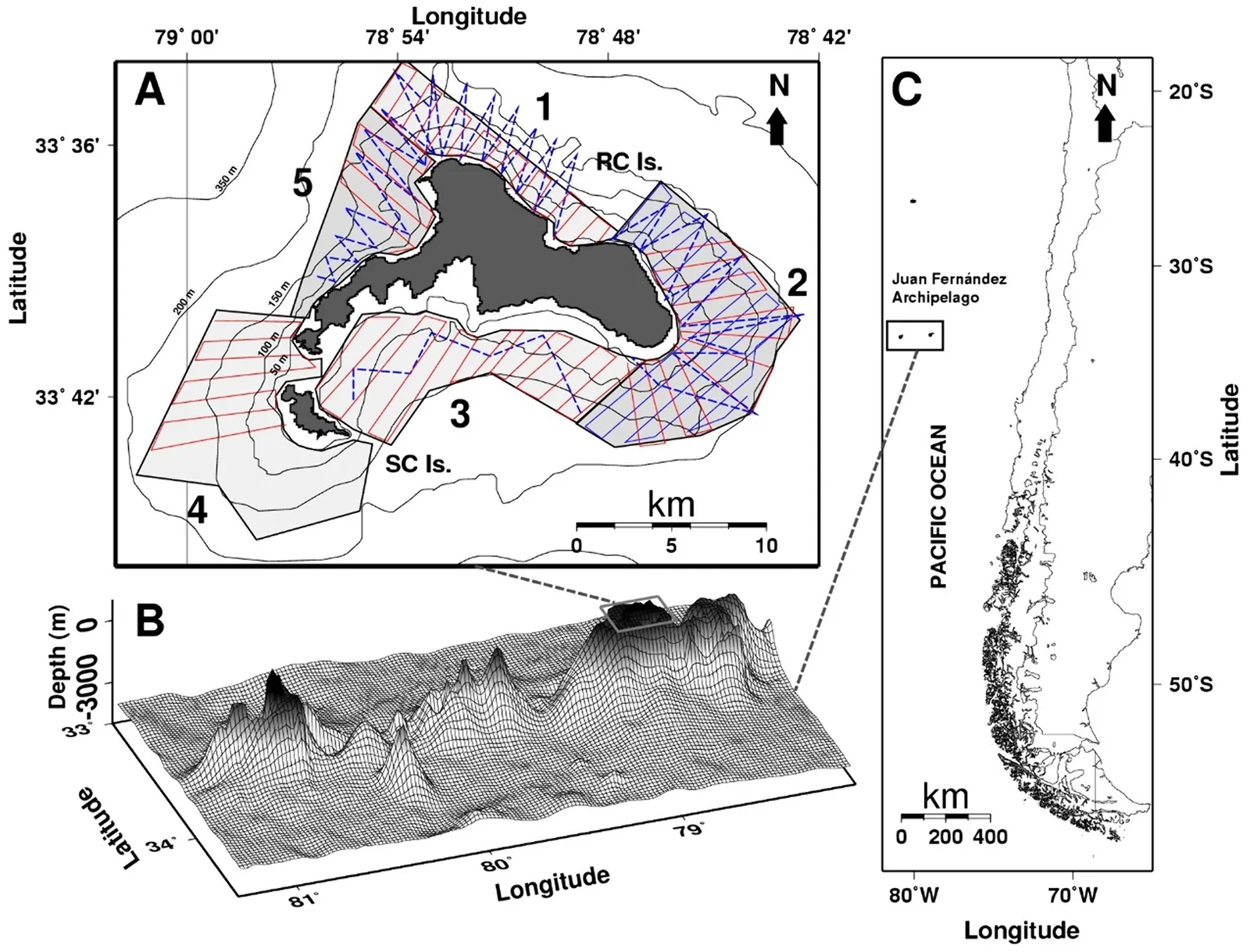
Geographic location of Juan Fernández Archipelago in the southeastern Pacific Ocean. (A) corresponds to the study site around Robinson Crusoe–Santa Clara Islands. Numbers (1–5) indicate the zones defined for hydroacoustic sampling. Thin blue lines within the polygons correspond to 2015 (dashed) and 2016 (red continuous) hydroacoustic transects. (B) depicts the Juan Fernandez Ridge, from which emerge the Robinson Crusoe–Santa Clara and Alejandro Selkirk Islands. (C) depicts the southeastern Pacific. The map was created by the authors using GMT v6 [10] and bathymetric data from NOAA NCEI (Public Domain: https://data.ngdc.noaa.gov/ngdcinfo/privacy.html).

Geomorphological features (e.g., reefs, bathymetry), remote/local oceanographic processes, and biophysical interactions on the shelf have been shown to determine species distribution patchiness [19–23]. While rockfish communities are often used as an example, showing strong correlations among abundance, aggregation levels, and geomorphological reef features [7,24,25], other studies have also demonstrated fish–reef associations across diverse ecosystems, linking reef structures with fish density, distribution, and biomass using hydroacoustic approaches [25–29]. In the JFA, aggregated multispecies demersal fish assemblages have been described in association with conspicuous reef systems of various sizes (Table 1; [14,15]). These reefs, in turn, are strongly correlated with traditional fishing grounds used by artisanal fishers around the islands [16,23].

**Table 1:** Main fish species comprising demersal assemblages detected by remotely operated vehicles and stereo cameras ([14,15]).

Scientific name	Common name	Local name
*Nemadactylus gayi* ^***e***^	Juan Fernandez morwong	Breca
*Caprodon longimanus*	Pink maomao	Graniento
*Callanthias platei* ^***e***^	Juan Fernandez splendid perch	Calantias
*Pseudocaranx chilensis* ^***e***^	Juan Fernandez trevally	Jurel de Juan Fernández
*Scorpis chilensis* ^***e***^	Juan Fernandez golden butterfish	Pampanito
*Hypoplectrodes semicinctum* ^***e***^	Juan Fernandez redbanded perch	Piloto de Juan Fernández
*Helicolenus lengerichi*	Rockfish	Chancharro
*Plagiogeneion* sp.	Juan Fernandez rubyfish	Colorado/Ojudo
*Seriola lalandi*	Amberjack	Vidriola/Palometa

Nine species stand out in terms of abundance on the insular shelf of the Robinson Crusoe–Santa Clara Islands, five of which are endemic (e).

For over a century, the JFA ecosystem has sustained a valuable small–scale artisanal fishery operating under a traditional territorial tenure system [30–32]. This traditional system has brought economic stability and social well–being to the Robinson Crusoe Island local community [33,34]. Despite the importance of fish communities to the JFA ecosystem and local economy, little is known about their conservation status, especially for morwong (locally known as breca, *Nemadactylus gayi*), an endemic species that supports artisanal fisheries and consumption [16]. Understanding the scales of natural variability in their distribution, behavior, and particularly their association with the seascape requires scientific surveys for evidence–based decision–making regarding the implementation of conservation (e.g., Marine Protected Areas [35]) and management plans for species of high commercial interest [36]. An essential step is thus to develop conservation plans and fishery management actions based on knowledge of the state of fish communities and their relationship with the seascape spatial heterogeneity [35], such as specific zones and depth with a significant presence of reefs and fish patches.

The study of fish communities with non–invasive and non–destructive sampling methods, such as hydroacoustic and optical technologies, can provide essential scientific information, even in hard–to–reach areas [25,37], while conserving the local ecosystem’s fragility. Previous studies in JFA were methodologically restricted to scuba diving surveys and, therefore, limited to shallower depths (<30 m). The focus of these studies was the taxonomic characterization of fish species, their association with the subtidal environment [38,39], and trophic ecology [40]. While these inventories provided valuable baseline information, advances in hydroacoustic methods have proven effective for characterizing seafloor habitats and linking geomorphological features with fish distribution and biomass in other ecosystems [28,41]. Multibeam and split–beam systems have been successfully applied to describe habitat complexity and its relationship with demersal fish communities, providing both abundance estimates and ecological information on the habitats that sustain fish populations [29]. This, in turn, improves the interpretation of backscatter patterns in relation to benthic structures [27, 28].

In this work, we use hydroacoustic technology to monitor deeper zones (up to 200 m) and to understand which features might influence the presence and acoustic backscatter of fish aggregations surrounding JFA [14,15]. Specifically, this study aims to characterize the morphology of the reef system on the Robinson Crusoe–Santa Clara Islands shelf and examine how reef height, length, location, and depth influence fish presence and acoustic backscatter, providing insights relevant to conservation and ecosystem–based management strategies.

## Materials and methods

### Study area and hydroacoustic surveys

The JFA comprises a group of volcanic islands around 6 million years old [42]. It is located perpendicularly to the southwestern edge of the South American continent, 360 nautical miles (nmi) to the west of the province of Valparaíso, Chile (Fig 1C; [12]). Three oceanic islands grouped into two subsystems form the JFA: Robinson Crusoe and Santa Clara Islands (RC–SC) and Alejandro Selkirk Island, ~ 90 nmi farther west (Fig 1B; [43]).

In this study, data were obtained from two daylight hydroacoustic surveys conducted around RC–SC subsystem in 2015 and 2016 (Fig 1A). Sailing permits for these operations were granted by the Chilean Navy within the JFA. Each survey had its own sampling designs [14,15]. The first survey was conducted between November 6th and 9th, 2015, using a 12–m diesel–powered vessel at a navigation speed of 4–6 knots. SIMRAD EK60 split–beam transducers of 38 and 120 kHz (ES38–B and ES120–7C; Kongsberg Simrad AS, Kongsberg, Norway) were installed on a deployable metal arm two meters below the surface. The downward–looking echosounders were calibrated following standard procedures [44] and configured with a pulse duration of 1024 µs and a ping rate of 1.0 s^-1^. Sampling followed a zig–zag design with transects of 4.6 km long separated approximately by 1.1 km between apices (Fig 1A). This sampling strategy was developed as part of a 2015 exploratory monitoring project to understand the distribution and composition of the fish community on the RC–SC Islands coastal marine shelf [14] using hydroacoustic and optical sampling techniques.

The data on the distribution and composition of the fish aggregations from the first survey allowed us to refine the sampling design on the second survey, which presents a more precise sampling design, bound to the coastal fish species distribution (Table 1). The second survey was conducted on a 9–m artisanal fishing boat (with the same navigation speed of 4–6 knots as in the previous survey) between October 6th and 12th, 2016. In this case, only the 38 kHz echosounder was used, with an ES38–10 transducer model, maintaining the same configuration as the 2015 study but mounted in a tow–body suspended off the vessel’s side and deployed one meter below the surface. The sampling design implemented quasi–parallel (4.3 km long) transects, configured orthogonally to the coast, and spaced 0.93 km apart.

Both survey designs correspond to sampling strategies traditionally used for insular ecosystems [37]. In both surveys, the sampling area was stratified into five zones around the islands (Table 2 and Fig 1A). The zone selection was based on the captain/fishermen’s knowledge of fishing grounds, which are characterized by particular wind and wave conditions generating less or more sheltered zones for the sampling based on prevailing meteorological conditions.

**Table 2:** Geographic and operational characteristics of hydroacoustic survey zones.

Year	Sampling Date	Zone	Zone ID	Zone area (km^2^)	Number of transects	Total distance sampled (km)	Total area sampled (km^2^)	Proportion of zone surveyed
**2015**	6 – Nov	Puerto Inglés	1	27.60	14	40	0.560	0.018
	7 – Nov	Tres Puntas	5	21.16	15	26	0.360	0.017
	8 – Nov	El Verdugo	2	52.74	12	39	0.544	0.010
	9 – Nov	Puerto Inglés	1	27.60	14	25	0.344	0.012
**2016**	6 – Oct	Tres Puntas	5	21.16	5	21	0.429	0.020
	7 – Oct	Santa Clara	4	25.98	7	36	0.804	0.031
	8 – Oct	El Verdugo	2	52.74	11	61	1.430	0.027
	9 – Oct	Puerto Inglés	1	27.60	16	28	1.890	0.068
	10 – Oct	Playa Larga	3	45.15	13	53	3.428	0.076
	12 – Oct	El Verdugo	2	52.74	12	55	3.753	0.071

Surveyed areas around the Robinson Crusoe–Santa Clara Islands during the 2015 and 2016 hydroacoustic surveys. Zone areas (km^2^) were calculated using the geographic processing software ArcGIS.

### Acoustic data processing

Acoustic records at 38 and 120 kHz obtained during the 2015 survey were initially used to qualitatively discriminate the acoustic signal of fish assemblages from zooplankton sound scattering based on backscatter intensity, aggregation shape, depth, and location in the water column. However, only the 38 kHz frequency was used quantitatively in this analysis because acoustic records were available for both the 2015 and 2016 surveys. The use of this frequency is further supported by its reliability in deeper waters, because at ~200 m depth–related attenuation and changes in swim bladder target strength are minimal (< 3 dB; [37,44]), ensuring consistent detection of fish schools [37].

Data was processed using Echoview® 8.0.105 acoustic analysis software [45]. Acoustic backscatter processing involved configuring the survey transects’ start and end, removing transect sections located outside the 5 zones established around the islands, as well as intertransect data, and delineating the water column from 7 m below the surface to 1 m above the detected seafloor. The upper limit was set to exclude surface noise, e.g., bubbles from waves and other disturbances, while the lower limit avoided bias from the near–bottom echoes (where discrimination from reflected ocean floor can be problematic) and potential side–lobe interference. Discrete aggregations of fish with discernible edges were identified along transects using a threshold in volume backscattering strength (Sv; [46]) of −70 dB re 1 m^-1^ to create user–defined regions (polygons) with assigned names (IDs). We separated multispecies demersal fish aggregations from pelagic ones based on morphology, depth, and the aggregations’ positions in the water column. These aggregation types were identified and characterized through optical sampling [14,15]. Underwater camera systems (ROV and stereo cameras) used during 2015 on echo traces indicated that fish aggregations located in midwater and within the upper layer of the water column were constituted mainly by Juan Fernández trevally, while the fish aggregations linked to the seafloor (forming columnar and bottom associated aggregations) were composed of mixed species [14,15], which constitutes the focus of this study (Table 1).

The integration of fish acoustic backscatter values per unit area “NASC” (Nautical Area Scattering Coefficient, units of m^2^ nmi^-2^; [46]) was exported in Elementary Distance Sampling Units (EDSUs) covering 50 m along each transect, spanning the entire water column. NASC represents the sum of volume backscatter (Sv) within an area and is proportional to the biomass or abundance (number of individuals) and type of organisms producing this backscatter [37,44]. Although NASC does not directly depict absolute fish density, it is also used as a proxy of acoustic density, i.e., a relative fish density indicator. For each EDSU, acoustic backscatter of fish aggregations and its subsequent transformation into presence–absence data, bathymetry, and geographic location were recorded.

The characterization of a reef’s shelf entailed the identification of features indicated by significant variations in bathymetric relief and acoustic backscatter intensity observed in analyses of bottom echoes (see Fig 2). Reefs were isolated within the echograms by selecting locations (delimited by regions and IDs) where the bottom height was conspicuously above the average seafloor height (>2m), measured from a manually projected baseline from the detected seafloor, as illustrated in Fig 2B. Based on these measurements, a reef’s presence–absence, reef mean height, length, and depth attributes were extracted for each EDSU. Importantly, these attributes were only measured when reefs were present; EDSUs without reefs were not assigned values for these variables. Region–specific IDs were retained when integrating these features across transects to account for fish aggregations and reefs spanning more than one EDSU.

**Fig 2:**
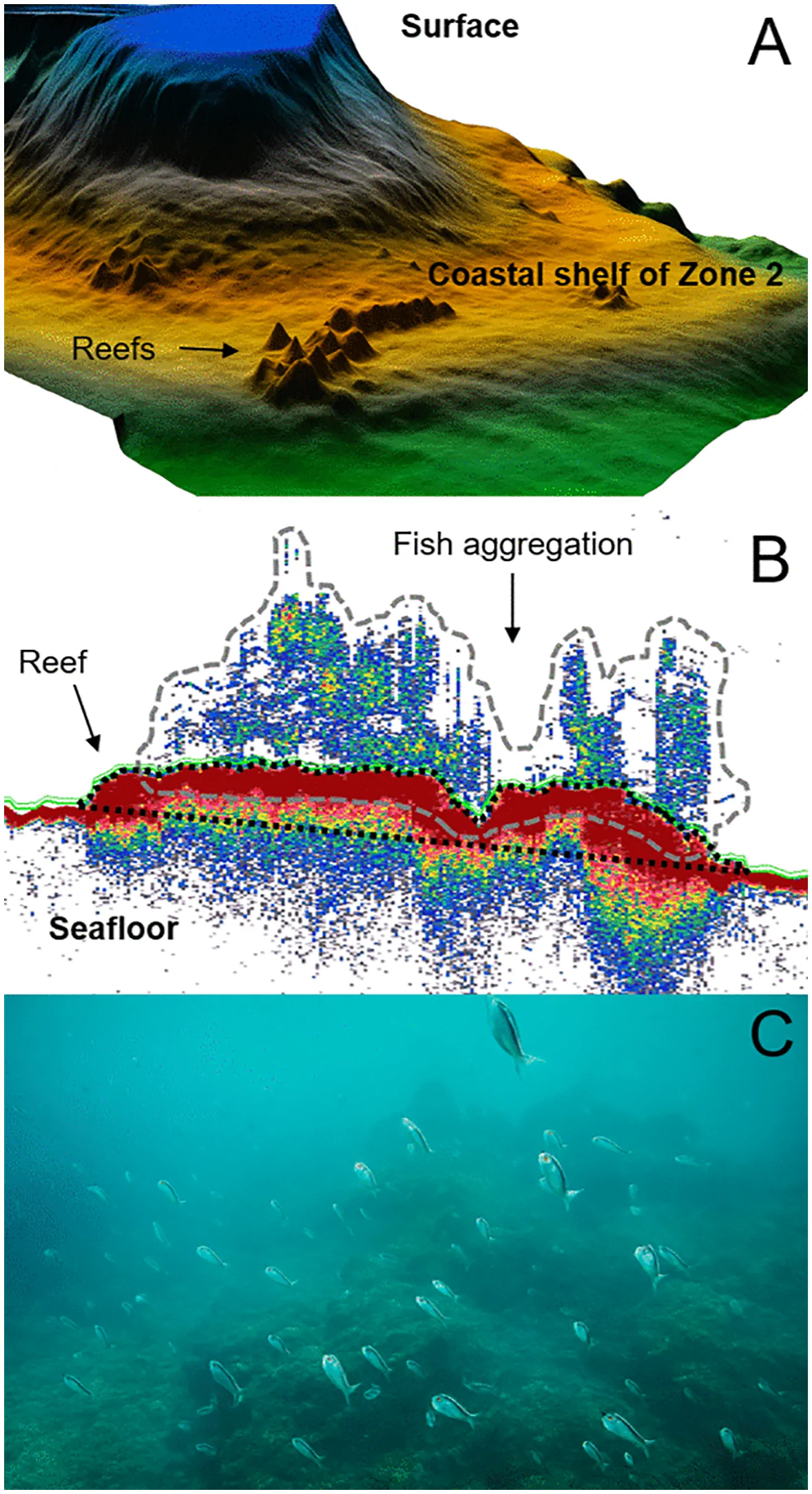
Reefs and fish aggregations on the insular shelf. (A) Bottom profile of the southeastern insular shelf of Robinson Crusoe Island, showing temperate reefs and reef aggregations. (B) shows an echogram of fish aggregations and temperate reefs of this region recorded during the 2016 hydroacoustic survey, and (C) a fish school depicted from stereo cameras (see Ernst et al., 2016 for further details).

#### Characterization of rocky reef structural attributes.

Four attributes were used to characterize rocky reef structure: average reef height, total reef length, geographical locations (zones), and reef depth stratum. The average reef height was calculated by averaging all height observations within each EDSU, with the reef’s baseline defined as a projected straight line based on bathymetric slope, as shown in Fig 2B. The total reef length was calculated from the Euclidean distance between the initial and final geographic coordinates of the identified reef from the echograms (Fig 3). The reefs were also geographically referenced to the five zones around the RC–SC Islands (Fig 1A) defined in section 2.1. Finally, the reefs were classified into four depth strata across the shelf: 0–50 m, 50–100 m, 100–150 m, and 150–200 m.

**Fig 3:**
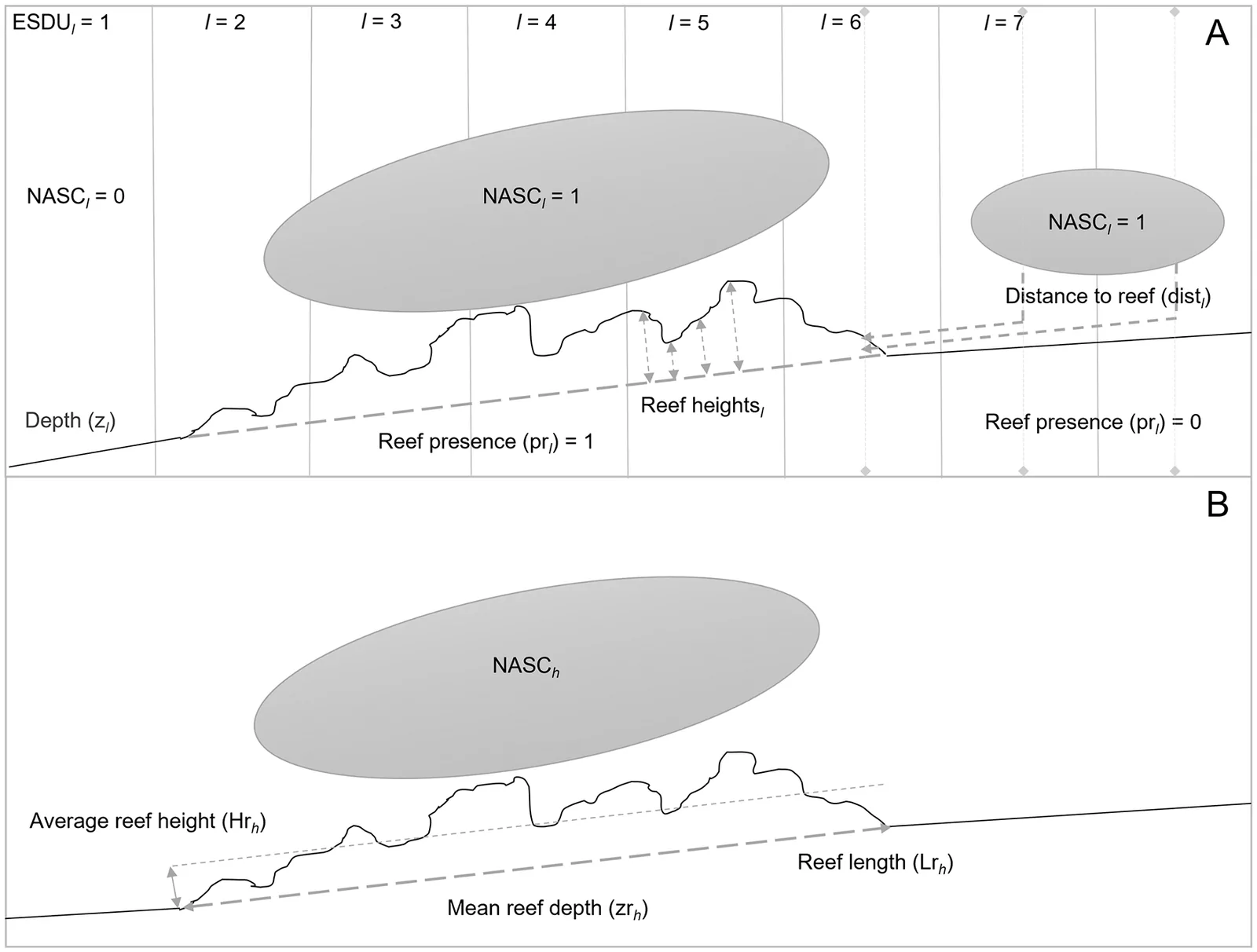
Schematic representation of reef and fish aggregation metrics at different scales. Conceptual diagrams of reef and fish aggregation metrics used during the post–processing and (A) analysis of acoustic data at the Elementary Distance Sampling Units (EDSU) of 50 m and (B) reef scales.

#### Spatial distribution of rocky reefs.

Survey and reef coverage areas based on acoustic backscatter data were used to obtain spatial rocky reef statistics computed by zone and depth, such as average reef density (number of reefs/ km^2^), proportion of reef area coverage, and expected number of reefs.

Given that transects were generally inshore–offshore oriented and beam width varies with depth, a trapezoid was used to estimate the areas. Acoustic beam diameters (trapezoid bases) were calculated using transducer beamwidth and local bathymetric depths, where nominal beamwidths at half–power points (ES38–B and ES38–10) corresponded to 7 and 10 degrees on hydroacoustic surveys in 2015 and 2016, respectively. The Euclidean distance between initial and end coordinates was used to estimate the surveyed distance or reef’s length (trapezoid height). These measurements (diameters for trapezoid bases and length for height) were combined to calculate the survey transect (*a*) and the reef coverage (*r*) areas.

The reef density was estimated from the amount of reef detected in each transect segment (*s*_*i,j,k*_) within zone ‘*i’*, depth stratum ‘*j’* and transect ‘*k’*. Transect segments (*s*_*i,j,k*_) were defined based on the 50 m EDSUs and could include one or more EDSUs, or a portion thereof, within the same zone and depth stratum. This approach ensures that reef densities account for all observed reefs and the effective survey area, while also accommodating differences in transect length across zones and depth strata. The densities (*d*_*i,j,k*_) were then weighted by the proportion of surveyed area $\left(\frac{a_{i,j,k}}{\sum_{k=\text{1}}^{{ns}_{i,j}}a_{i,j,k}}\right)$, reflecting variation in transect lengths due to distance to depth contours (isobaths), where ‘*ns*_*i,j*_*’* is the number of transect segments by zone and depth stratum. The average reef density by zone and stratum, ($\hat{d_{i,j}}$) was calculated as:


$$\begin{array}{c} \hat{d_{i,j}}=\frac{\sum_{k=\text{1}}^{ns_{i,j}}\left(d_{i,j,k}\times a_{i,j,k}\right)\;}{\sum_{k=\text{1}}^{ns_{i,j}}a_{i,j,k}\;} \end{array}$$ (1)

The proportion of reef area coverage by zone and depth stratum *(*$\hat{{Pr}_{i,j}}$*)* was obtained through the ratio of total reef area coverage ($\sum_{k=\text{1}}^{ns_{i,j}}r_{i,j,k}$) to the total sampled area ($\sum_{k=\text{1}}^{ns_{i,j}}a_{i,j,k}$):


$$\begin{array}{c} \hat{{Pr}_{i,j}}=\left(\frac{\sum_{k=\text{1}}^{ns_{i,j}}r_{i,j,k}}{\sum_{k=\text{1}}^{ns_{i,j}}a_{i,j,k}}\right) \end{array}$$ (2)

Finally, the number of reefs ‘*e’* by zone and depth stratum,$\left(\hat{Ee_{i,j}}\right)$ was estimated based on average density estimates (*d*_*i,j*_) and the total area (*A*_*i,j*_), assessed using the geographic processing software ArcGIS (Environmental Systems Research Institute, ESRI, Table 2). This approach assumes approximate homogeneity of reef density within each depth stratum and zone (*d*_*i,j*_).


$$\begin{array}{c} \hat{{Ee}_{i,j}}=d_{i,j}\times A_{i,j} \end{array}$$ (3)

### Analysis of demersal fish assemblage data

The analysis of demersal fish data consisted of modelling both the presence–absence of fish aggregations (a discrete response variable) and their acoustic backscatter (a continuous response variable).

#### Discrete response variable: presence–absence of fish aggregations.

A binomial generalized additive model for location, scale and shape (GAMLSS; [47,48]) implemented in R [49] was used to explore, by EDSU ‘*l*’, the effect of several factors on the probability of occurrence (*NASC*_*l*_ = *1*) of demersal fish aggregations (*n*_*EDSU*_ *= 7462*). The primary explanatory variables included the presence–absence of reefs (*pr*_*l*_), a non-linear P–spline smoother function (S; [50,51]) on the seafloor depth (*z*_*l*_), and the distance to the closest reef (*dist*_*l*_; Fig 3A):


$$\begin{array}{c} og\left(p_{l}/(\text{1}-p_{l})\right)=\beta_{\text{0}}+\beta_{\text{1}}pr_{l}+S_{\text{2}}{(z}_{l})+{\beta_{\text{3}}dist}_{l} \end{array}$$

$$\begin{array}{c} {NASC}_{l}\sim Bernoulli\left(p_{l}\right);\text{0}\leq p_{l}\leq \text{1} \end{array}$$ (4)

A stepwise model expansion approach was used to test spatial heterogeneity around the RC–SC Islands coastal shelf based on the full dataset. A series of models was evaluated, beginning from a baseline framework (*M0*) that included all primary predictors, to a fully saturated model (*M4*) by a stepwise inclusion of zone as a fixed factor and its interactions with the predictors (*pr*_*l*_ *× zone,* S*(z*_*l*_*) × zone,* and *dist*_*l*_ *× zone*). To mitigate potential biases and overfitting in the full model [52], an iterative approach was utilized to evaluate various combinations of predictors, ensuring an optimal balance between model accuracy and complexity. The most parsimonious model obtained from this procedure was referred to as *M5*, selected using the stepAIC() function from the MASS package [53]. The predictive performance of models was compared using Akaike’s information criterion (AIC; [54]), with lower values indicating better model fit [55]. Additionally, the relative importance of each predictor within each zone was assessed through simple regression models with specific spatial interaction term, estimating the proportion of explained variance via squared Spearman rank correlation as a proxy for localized variance explained.

During the 2015 and 2016 hydroacoustic surveys, zones 1 and 2 were the most revisited of the zones (Table 1). This allowed isolation of a spatial–temporal subset to explore the consistency of the presence of demersal fish aggregations across these zones over time (season: 2015–2016). Spatial and temporal consistency was tested by contrasting a baseline model with season as an additive predictor (*ST*_*0*_) against a spatial model featuring triple interaction terms among the linear primary predictors, zones, and survey season (*ST*_*1*_*; zone × {pr*_*l*_*,* S*(z*_*l*_*), dist*_*l*_*} × season*).

#### Continuous response variable: Backscatter (NASC) of fish aggregations.

To identify the reef (*h*) structural attributes that may affect the relative acoustic density of fish, a reef–scale analysis was implemented. Contiguous EDSUs with reefs and fish aggregations were integrated by matching specific IDs assigned to each school and the reef polygons (*n = 276*) described in section 2.2. The integrated fish acoustic density value per unit area was modeled as a continuous variable (NASC of fish aggregations) through a GAMLSS with Weibull distribution, which was determined to be the optimal error structure via the fitDist() function. The response variable was modeled as a function of three reef attributes, namely mean depth (*Zr*_*h*_), length (*Lr*_*h*_), and average height (*Hr*_*h*_; Fig 3B). All of the explanatory variables were fitted using nonparametric P–spline smoother function:


$$\begin{array}{c} og\lambda_{h}=\;\beta_{\text{0}}+S_{\text{1}}(Zr_{h})+S_{\text{2}}{(Lr}_{h})+S_{\text{3}}({Hr}_{h}) \end{array}$$

$$\begin{array}{c} {NASC}_{h}\sim Weibull\left(\lambda_{h},k\right);y>\text{0},\lambda >\text{0},k>\text{0} \end{array}$$ (5)

The NASC response variable was modeled using a stepwise regression framework. Beginning from a baseline model without spatial structures (*M6*) that integrated reef depth, length, and height, the model was subsequently saturated by incorporating the interaction between reef attributes and zones (*M10*). An iterative procedure analogous to that used for the presence–absence models was employed to determine the optimal multiple predictor combinations (*M11*). Additionally, simple regression models with zone–specific interactions were implemented to identify the local relative importance of each reef attribute within individual zones, using the proportion of explained variance as a proxy.

## Results

### Spatial heterogeneity of the rocky reefs

#### Characterization of rocky reefs around the islands.

Bathymetric features of different sizes were found on the coastal shelf of RC–SC Islands. The reefs’ length varied between 14 and 985 m, and height ranged from 2 to 28 m (Fig 4A). Seventy–five percent of the reefs were under ~328 m long and ~9 m high (Fig 4B and 4C). Very long reefs (>760 m) were observed in zones 2, 3, and 4 (Fig 5A), mainly in deep strata 100–150 and 150–200 m (Fig 5B), as well as very high ones in zone 1 (>20 m; Fig 5C) associated with intermediate depths (50–100 and 100–150 m; Fig 5D). These features are observed as discrete spikes in the density contour of reef length and height (Fig 4A).

**Fig 4:**
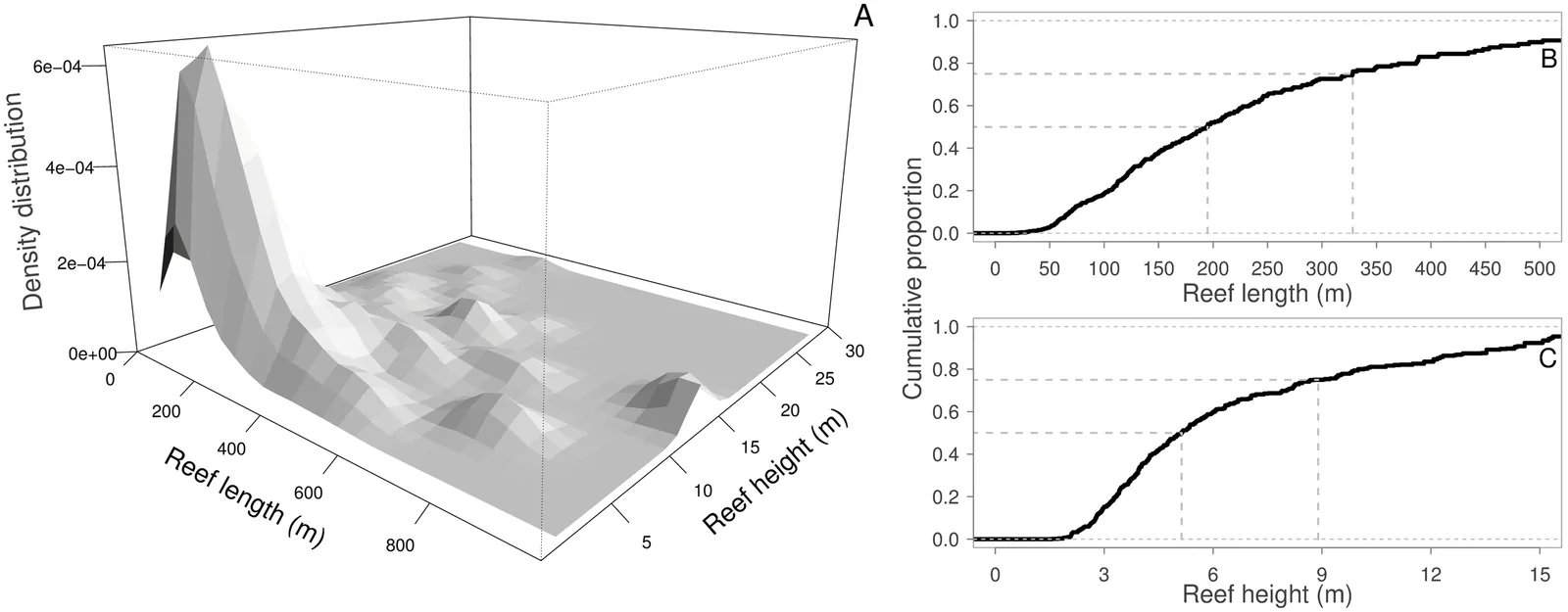
Reef length and height distribution. (A) Density distribution of reef length and height. (B) Cumulative proportion of reef length and (C) height. Dashed lines correspond to the 0.5 and 0.75 percentiles.

**Fig 5:**
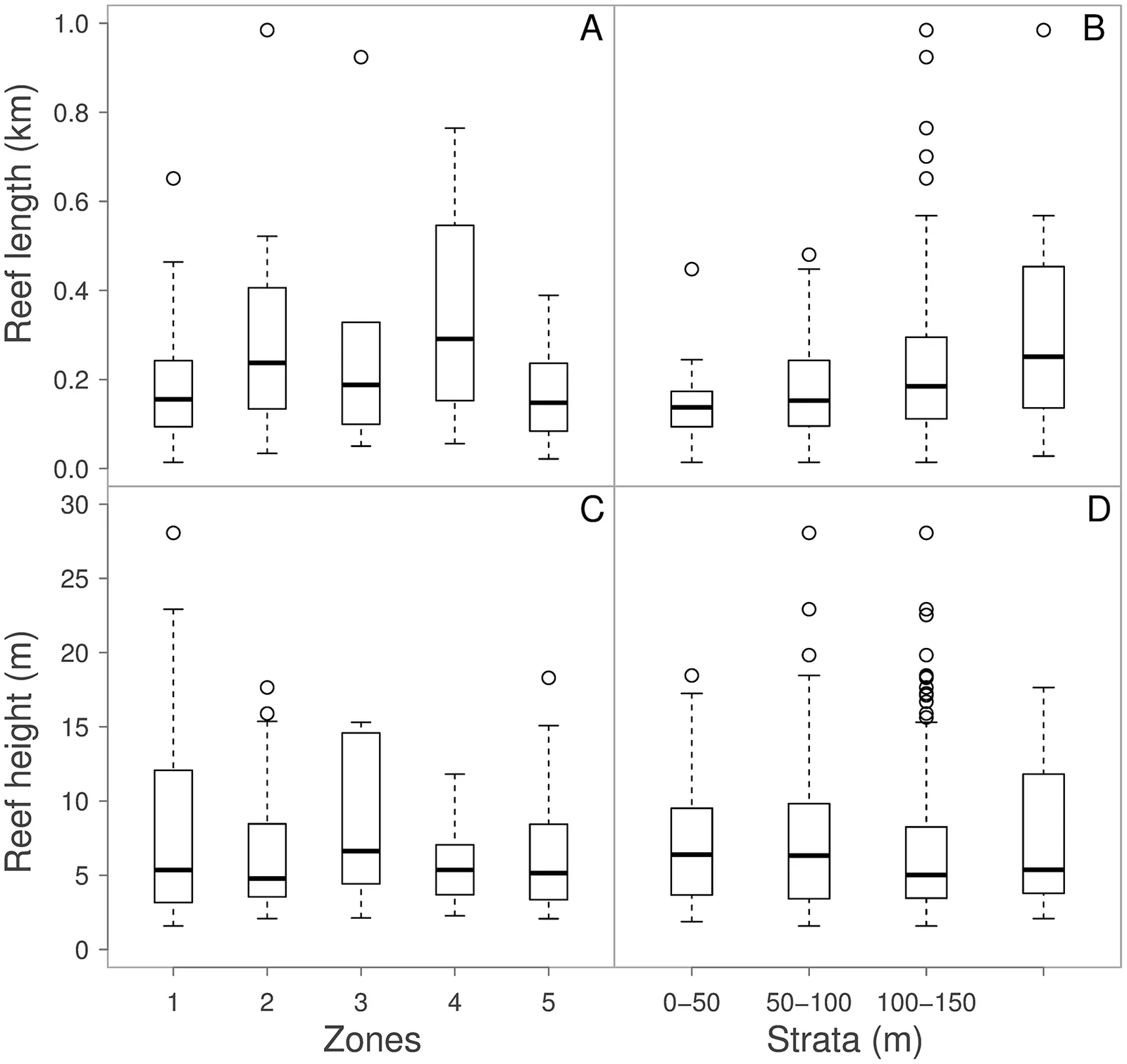
Reef morphometrics by zones and depths. (A) Boxplots of reef length by zones (n = 5) and (B) depth strata (n = 4). Panels (C) and (D) depict reef height by zone and depth strata, respectively, around the Robinson Crusoe and Santa Clara Islands.

#### Spatial distribution of structural rocky reef attributes around the islands.

High densities of reefs were observed in zones 1, 4, and 5 (>100 reefs/ km^2^; Table 3). Zone 4 had the largest proportion of reef area among the five zones (Table 4), mainly due to larger reefs (length, Fig 5A and S1A). Zones 1 and 5 also exhibited a high proportion of reef areas (Table 4), but this was largely due to the density of reefs rather than their size (Table 3 and S1A Fig). Reef length was the lowest in these zones than in any other (Fig 5A and S1A). Reef height was highest in zones 3, 4, and 1 (Fig 5C).

**Table 3:** Assessing reef density around Robinson Crusoe and Santa Clara Island.

	Zone					
Depth strata (m)	1	2	3	4	5	Mean
**0–50**	397	124	36	420	657	327
**50–100**	112	20	15	91	127	73
**100–150**	45	20	7	19	26	23
**150–200**	7	10	0	17	72	21
**Mean**	140	44	14	137	221	

Mean reef density (number of reefs / km^2^) by zone and depth strata around the Robinson Crusoe–Santa Clara Islands during the 2015 and 2016 hydroacoustic surveys.

**Table 4:** Reef coverage across zones and depths.

	Zone					
Depth strata (m)	1	2	3	4	5	Mean
**0–50**	0.153	0.103	0.012	0.095	0.156	0.104
**50–100**	0.163	0.018	0.026	0.332	0.144	0.136
**100–150**	0.087	0.066	0.025	0.259	0.090	0.105
**150–200**	0.013	0.054	0.000	0.228	0.010	0.061
**Mean**	0.104	0.060	0.016	0.228	0.100	

Proportion of sampled area covered by reefs (reef area /sampled area) by zone and depth strata during the 2015 and 2016 hydroacoustic surveys.

#### Spatial distribution of structural rocky reef attributes by depth strata.

The average reef density was higher in the shallow (0–50 m) and intermediate strata (50–100 m), with around 300 and 70 reefs/ km^2^, respectively, decreasing towards deeper zones to ~20 reefs/ km^2^ (Table 3 and S1B Fig). Reef height exhibited a similar pattern (Fig 5D), while reef length displayed an opposite trend, increasing with depth (Fig 5B and S1B). The highest proportion of reef area was observed in the shallow and intermediate strata, 0–50, 50–100, and 100–150 m (Table 4 and S1B Fig). Spatial differences in the reef area were affected by reef density and length. In fact, 0–50 m has the greatest density of small reefs, 50–100 m contained relatively small reefs, but it had the second largest density, while 100–150 m hosted the third largest density, but the second stratum with the longest reefs (<150–200 m; Table 3; Fig 5B and S1B).

#### Number of expected reefs by depth strata and around the islands.

The expected number of reefs was obtained by weighting the averaged reef density by the area of each depth stratum, showing a predominance of reefs at shallow and intermediate strata (0–50, 50–100, and 100–150 m, ~ 6587 reefs), with zones 1 and 5 making the highest contribution (>2000 reefs each). The northeastern and northwestern regions (zones 1 and 5) accounted for 61% (~4501 reefs) of the total number of reefs on the insular shelf (~7351; Table 5).

**Table 5:** Number of reefs on the insular shelf.

	Zone					
Depth strata (m)	1	2	3	4	5	Sum
**0–50**	943	282	56	627	1213	3121
**50–100**	637	160	173	345	535	1850
**100–150**	517	441	179	198	281	1616
**150–200**	56	211	0	178	319	764
**Sum**	2153	1094	408	1348	2348	

Number of reefs by zone and depth strata during the 2015 and 2016 hydroacoustic surveys.

### Linking rocky fish assemblage and reefs

Reef habitats comprised approximately 9% of the total area surveyed, however, empirical cumulative distribution analysis showed that half of the fish aggregations were located within 48 m of the nearest rocky reefs, and 75% within 280 m (Fig 6). This spatial clustering highlights the strong association between fish aggregations and rocky reefs on the RC–SC insular shelf.

**Fig 6:**
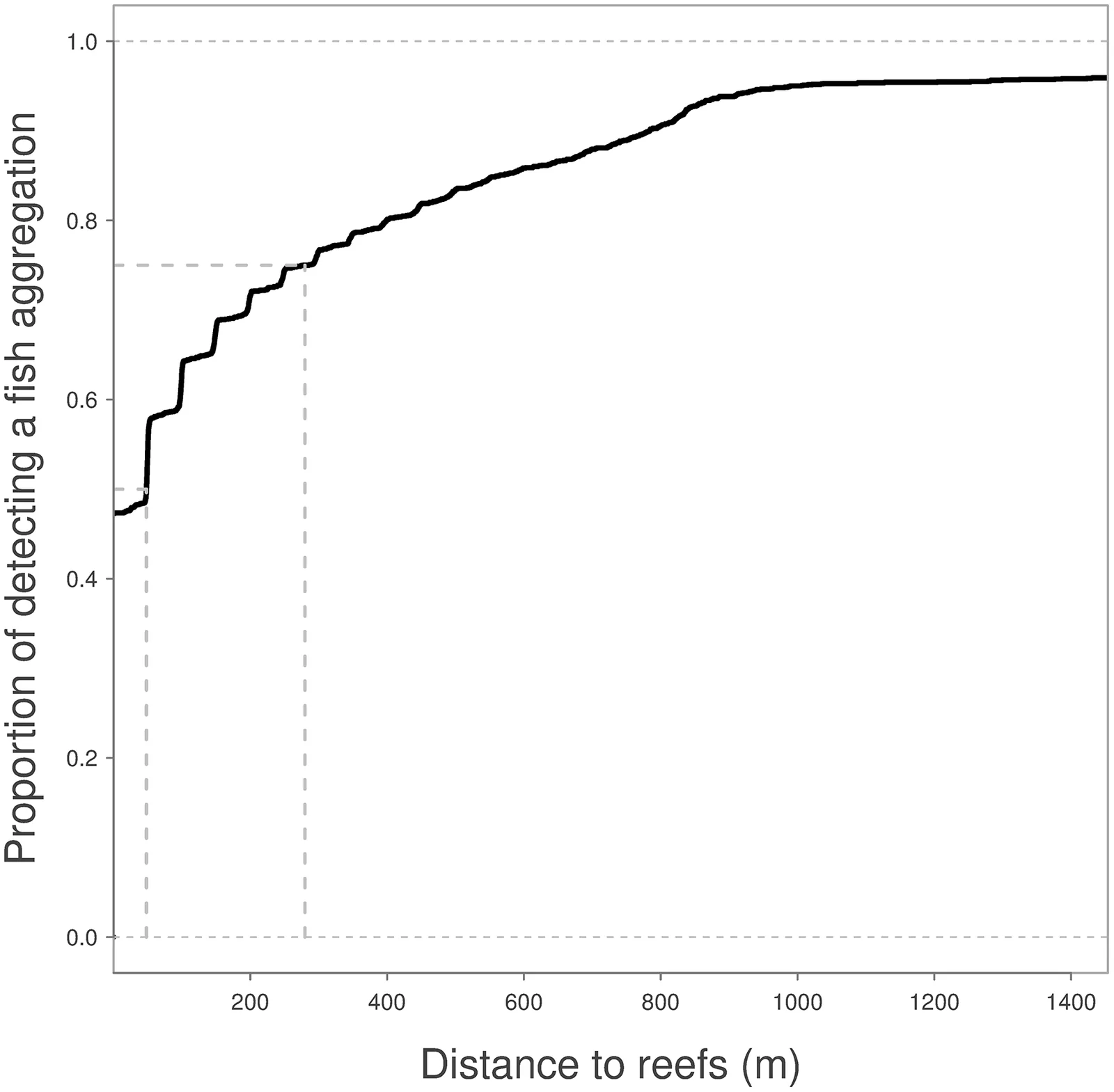
Fish detection as a function of reef distance. Empirical cumulative proportion of observing fish aggregations (a discrete variable) as a function of the distance to the nearest reef. Dashed vertical lines correspond to the 0.5 and 0.75 percentiles.

#### Modelling discrete response variable: presence–absence of fish aggregations.

The multiple regression analysis showed that the relationships between the presence of demersal fish aggregations and habitat predictors were strongly heterogeneous across the RC–SC Islands coastal shelf. Allowing habitat predictors to interact with specific spatial zones significantly improved model parsimony, as the sequential addition of zone interactions with reef presence (*pr*_*l*_), seafloor depth (*z*_*l*_) and distance to the closest reef (*dist*_*l*_) reduced 527 AIC units compared to the baseline model (*M0*; see S1 Table). Consequently, the stepwise selection identified *M5* as the optimal model (AIC = 6061; S1 Table), preserving the habitat–zone interactions except for the seafloor depth effect in zone 5 (S2 Table).

Across all zones combined, model 5 (*M5* full model) indicated that, in the presence of reefs, there is a ~ 0.9 probability of finding demersal fish aggregations at depths up to ~150 m, dropping to its lowest levels at greater depths and approaching ~0.4 at 200 m. When reefs were absent, the probability of detecting demersal fish aggregations was substantially lower (~0.2–0.3) at depths up to ~150 m and declined outside of this range, following a dome–shaped pattern that reflects the smoother function depth effect’s significance (Fig 7A). This pattern highlights a key depth range for aggregations. The sharp decline in the probability of detecting fish aggregations in areas with and without reefs within the 150–200 m depth range indicates that deeper zones are likely outside the preferred demersal habitat.

**Fig 7:**
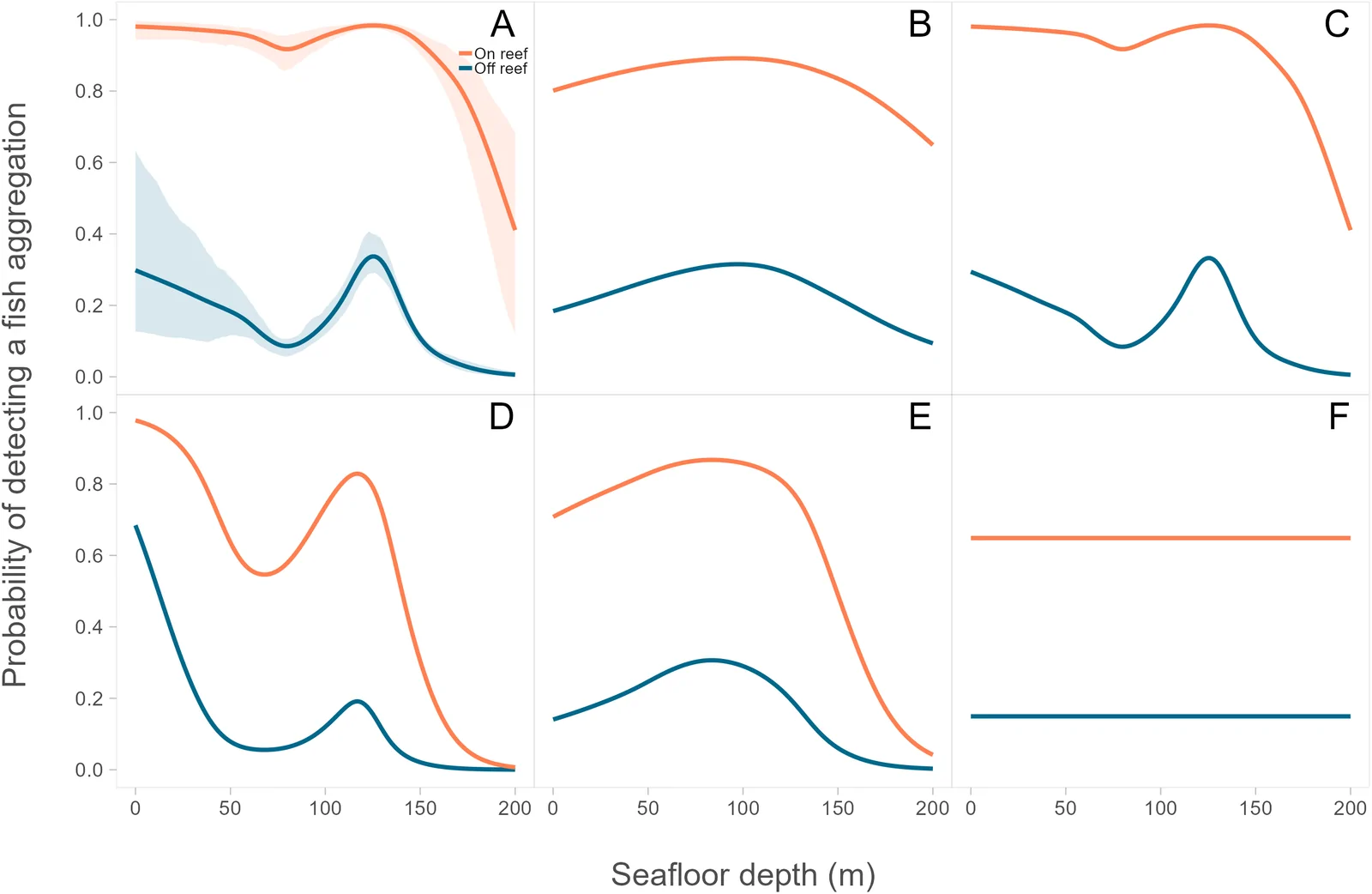
Spatial heterogeneity of fish detection probability as a function of reef presence, seafloor depth, and distance to the nearest reef. Model predictions of the probability of observing demersal fish aggregations based on the full integrated spatial model (*M5*). Panel (A) represents the aggregated spatial prediction framework. The upper curves represent predictions over temperate rocky reefs (reef presence, distance to reef = 0 m), and the lower curves represent predictions outside reefs (reef absence, distance to reef = 579 m, the mean distance to the nearest reef when all zones were integrated, which may vary by specific zone). Shaded areas around the curves indicate the 95% confidence intervals. Panels (B–F) represent zones 1–5, respectively, which illustrate localized spatial responses shaped by site–specific bathymetric smoothing splines and interaction terms.

However, this ecological pattern exhibits notable local variations across specific areas. In zones 1 and 4 (Fig 7B and 7E), the curves depict the general dome–shaped bathymetric distribution, but the probability of detecting demersal fish aggregations is overall significantly lower (*zone*_*1*_: β= −0.649, *p* = 0.036; *zone*_*4*_: β = −2.13, *p* < 0.001; see S2 Table). Furthermore, these zones show a weaker positive effect of reef presence on fish detection, as evidenced by the negative interaction terms (*pr*_*l*_ *× zone*_*1*_*:* β= −1.873, *p* < 0.001; *pr*_*l*_ *× zone*_*4*_*:* β= −0.9, *p* = 0.022; S2 Table).

Conversely, zones 2 and 3 display significantly distinct non–linear curves. In zone 2 (Fig 7C), reef presence sustains a high, stable detection probability (~0.95) that extends to deep water before decreasing past 150 m (S*(z*_*l*_ *× zone*_*2*_*)*: β = −0.006, *p* = 0.002; S2 Table). In habitats without reef across zones 2 and 3 (Fig 7C and 7D), demersal fish detection probabilities drop to ~0.05–0.10 at intermediate depths (~75 m) before showing a significant increase at ~125 m (S*(z*_*l*_*)× zone*_*3*_: β = −0.027, p < 0.001; S2 Table).

Finally, zone 5 represents an ecological pattern that stands out because it shows a depth–independent relationship, where the stepwise selection criteria omitted the depth–smoothing predictor (S1 Table). Although the probability of detecting demersal fish aggregations within this zone is significantly lower overall (*zone*_*5*_: β = −2.15, p < 0.001; S2 Table), it exhibits constant probabilities across the entire bathymetric range, remaining fixed at ~0.65 over reefs and ~0.15 on habitats without reefs (Fig 7F).

The simple regression analysis with zone interactions isolated the local relative importance of each habitat predictor within individual zones (Table 6). These models showed that reef presence (*pr*_*l*_) was primarily predicted fish presence around the islands, except in zone 1, where the best predictor was distance to the closest reef (*dist*_*l*_; Table 6).

**Table 6:** Local relative importance of primary habitat predictors across zones.

Zone	EDSU (n)	Reef presence (*pr*_*l*_)	Seafloor depth (z_*l*_)	Distance to reef (*dist*_*l*_)	Dominant predictor
**1**	1918	23%	8%	32%	Distance to reef
**2**	2921	35%	12%	31%	Reef presence
**3**	1021	29%	7%	8%	Reef presence
**4**	678	31%	16%	26%	Reef presence
**5**	924	17%	5%	10%	Reef presence

For each zone, the relative importance of each predictor was computed through regression models with a zone interaction term. Values represent the estimated proportion of explained variance using the squared Spearman rank correlation as a proxy.

The spatial and temporal effect analysis for zones 1 and 2 tested whether the dome–shaped pattern described in Fig 7B and 7C holds across the 2015–16 survey seasons, using the fully interactive model (*ST*_*1*_: AIC = 4019; see S3 Table). Both zones maintained their general dome–shaped patterns in the probability of observing fish aggregations as a function of the bathymetric depth range, but exhibited significant temporal variations, with zone 1 (Fig 8A) showing a larger temporal contrast than zone 2 (Fig 8B). The interannual differences in zone 1 were primarily driven by a significant increase in the positive effect of reef presence during the 2016 season (*pr*_*l*_ *× season*_*2016*_*:* β = 1.695, *p* = 0.002; S4 Table). Conversely, zone 2 maintained highly stable, near saturated detection probabilities over reefs across both seasons, alongside a consistent localized peak at ~125 m depth in areas without reefs (S*(z*_*l*_*) × zone*_*2*_*:* β = −0.005, *p* = 0.014; Fig 8B and S4 Table).

**Fig 8:**
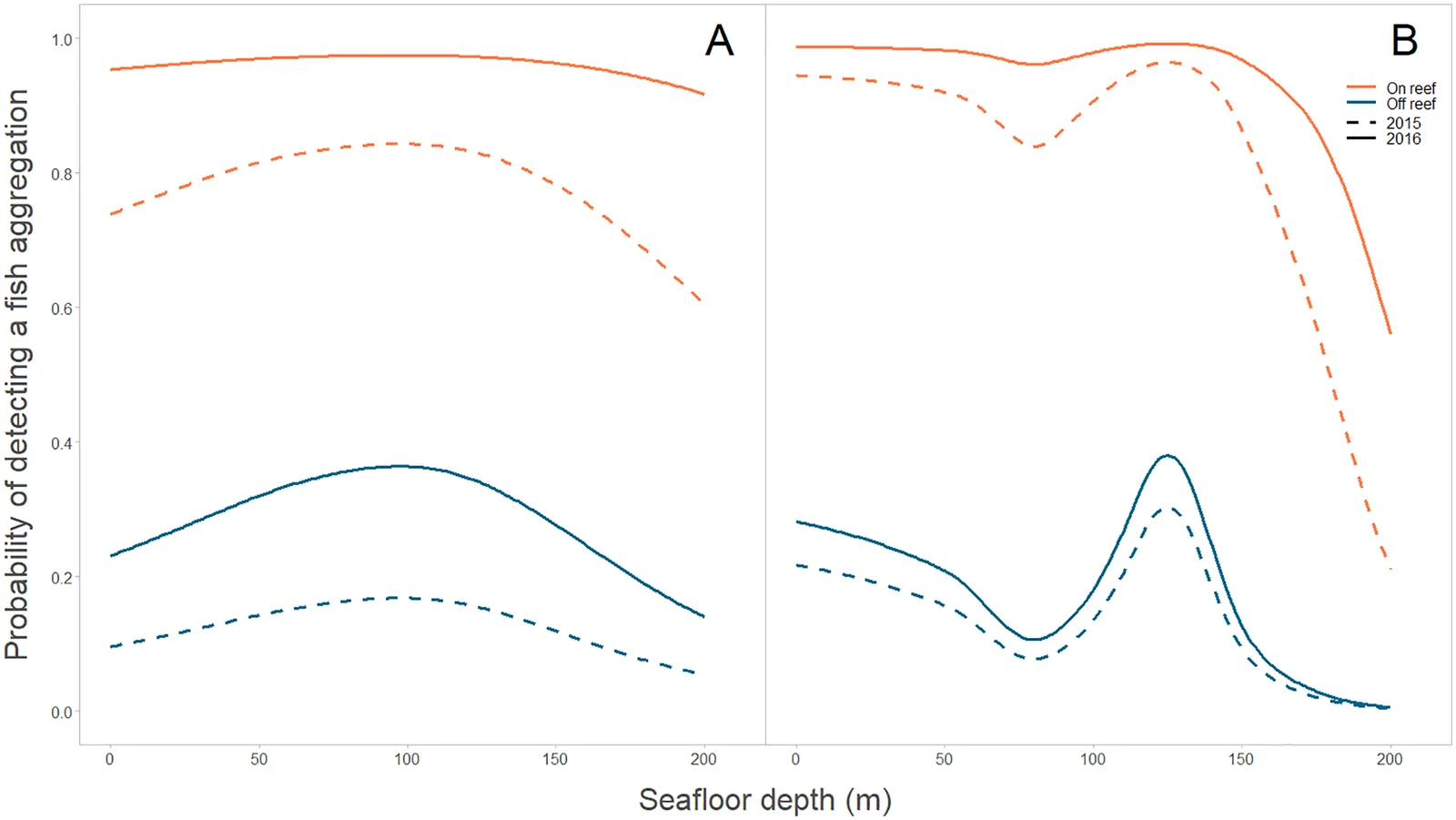
Spatial and temporal patterns in fish detection probability as a function of reef presence, depth, and distance to the nearest reef. Model predictions of the probability of observing fish aggregations based on interactive spatial and temporal model for (A) zone 1 (B), and zone 2. Colored lines represent predictions over temperate rocky reefs (distance = 0 m), and blue lines represent predictions outside them. Dashed lines indicate predictions for the 2015 survey season and solid lines represent predictions for the 2016 season.

#### Modelling continuous response variable: Backscatter (NASC) of fish aggregations.

In the multiple regression procedure, the fully integrated model with zone specific interactions *M10* (AIC = 4958; see S5 Table) showed the best fit to fish aggregations backscatter across the coastal marine shelf of the Juan Fernández Sea. This model indicated that shallower, longer, and taller reefs sustained significantly higher fish backscatter (Fig 9), though the magnitude of these effects varied per zone (S6 Table).

**Fig 9:**
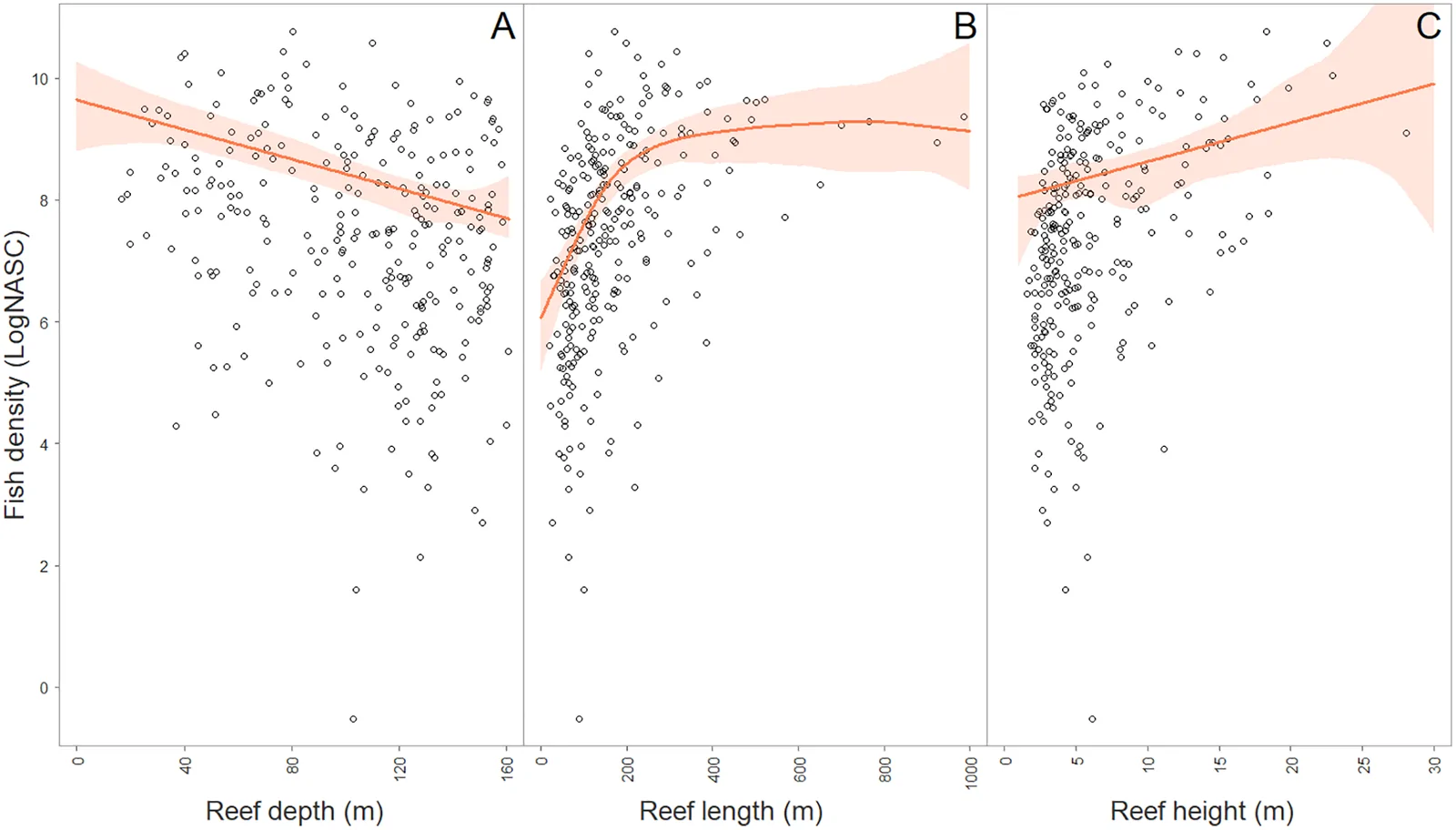
Fish backscatter (LogNASC) as a function of reef attributes. Model predictions of the backscatter of fish aggregations (logNASC) as a function of reef depth, reef length, and height, based on the main–effects baseline model *M6* (representing the mean population response across all zones; see S5 and S6 Tables for the best fitting interaction *M10*). In panel (A) fish backscatter was modeled as a function of reef depth (*Zr*_*h*_), using mean values of reef length (*Lr*_*h*_ = 170 m) and height (*Hr*_*h*_ = 6 m). In panel (B), predictions were made using mean values of reef depth (*Zr*_*h*_ = 104 m) and height. Panel (C) shows logNASC predictions as a function of reef height using mean values for depth and length. The orange shaded areas around the curves indicate 95% confidence intervals.

Reef depth exhibited a consistent and negative effect on fish acoustic density across all zones (Fig 9A), showing a general decrease that was particularly sharp in zones 3–5 (β ≤ −0.023, *p* < 0.001), and more moderate in zones 1–2 (β ≥ −0.011, *p* < 0.001; see S6 Table). Conversely, reef length had a significant positive effect on fish backscatter around the RC–SC Islands (Fig 9B), especially in zones 1–2 (β ≥ 0.006, *p* < 0.001), but not in zone 5 (*p* = 0.411; S6 Table). Similarly, reef height increased density across most of the islands (β ≥ 0.085, *p* ≤ 0.017; Fig 9C), except in zone 1, where the effect was non–significant (*p* = 0.529; S6 Table).

The simple regression analysis showed that, at the scale of individual reefs, fish backscatter was best explained by reef length (*Lr*_*h*_) in zones 1–2 (explained 35% and 29% of variance, respectively), average reef height (*Hr*_*h*_) in zones 3–4 (49% and 13%), and reef depth (*Zr*_*h*_) in zone 5 (28%; Table 7).

**Table 7:** Local relative importance of reef (*h*) structural attributes as predictors of fish backscatter (logNASC) across zones.

Zone	EDSU (n)	Reef depth (*Zr*_*h*_)	Reef length (*Lr*_*h*_)	Reef height (*Hr*_*h*_)	Dominant predictor
**1**	108	14%	35%	27%	Reef length
**2**	89	1%	29%	20%	Reef length
**3**	19	18%	39%	49%	Reef height
**4**	33	12%	3%	14%	Reef height
**5**	27	28%	23%	7%	Reef depth

As with discrete variable modelling, the relative importance of each predictor was computed using regression models with a zone interaction term using relative acoustic fish density (logNASC) as the response variable. Values represent the estimated proportion of explained variance.

## Discussion

The insular shelf of the Robinson Crusoe and Santa Clara Islands, a highly rugged seascape with numerous temperate reefs at different depths, constitutes a discrete, spatially heterogeneous environment vital for structuring populations and biological communities [7,26]. This research constitutes a first attempt to identify and highlight the ecological relevance of these rocky structures on the spatial distribution of local fish assemblages through the use of hydroacoustic sampling methods (down to a depth ~200 m). This work particularly contributes to identifying predictors of fish presence and relative density, which are of high interest for conservation plans and seascape ecological studies [56,57]. In addition, it addresses the need to develop monitoring and non–invasive, non–destructive survey programs that match this spatial seascape heterogeneity and provide robust information to strengthen future conservation plans [58].

### Reef characterization

The study of seafloor spatial heterogeneity has been approached by measuring reef density (number of patches along transects), size (height and length), and their spatial distribution on the insular shelf (zones and depth strata). Observing the same reef on multiple occasions, e.g., across neighboring transects, could lead to reef density overestimation. However, the observed maximum reef length was 985 m oriented offshore, while around 75% of the reefs were under ~328 m long and ~9 m high. The distance between transects was generally 926–1111 m in the 2015 and 2016 survey designs, respectively. Assuming a relatively symmetric distribution of reefs (relative to the shore), this source of error should be small.

Zones 1, 4, and 5 show a more rugged seafloor. In zones 1 and 5, this heterogeneity was due to the high density of narrow and tall reefs in a shallow, restricted shelf area (NE and NW; [11,13]). This region is less exposed (sheltered) to relevant erosion factors, such as the SW–prevalent wind and waves (Fig 10; [38]), which shape the reef’s geomorphology [56,59,60]. Whereas in zone 4, to the southwest of the shelf, the heterogeneity of the seafloor was mainly due to the large size of the reefs distributed over a deeper and broader area on the insular shelf (SW; [13]). Currents, sea level, winds, and waves are relevant agents of erosion that are typically more intense in the shallower areas [8]. This shallow–depth stratum from zone 4 corresponded to the SW shelf (<50 m depth), an area exposed to erosion and characterized by fewer reef patches than the sheltered regions (NE and NW). The insular shelf configuration, along with the reefs’ spatial distribution (highly correlated to fishing grounds [16,30,32]), has provided key information for the implementation of an effective fishery monitoring around islands (Fig 10). In the northern region (zones 1 and 5), our results suggest implementing sampling designs that cover a coastal and shallow area of the shelf (<100 m depth). While in the southern region (zone 4), a sampling design that is less coastal and deeper is necessary outside of the breaker areas (>50 m depth). Coincidentally, the southern shallow area is characterized by high intensity of winds and waves [38], posing operational constraints to the finfish artisanal fleet [16] and for research vessel navigation.

**Fig 10:**
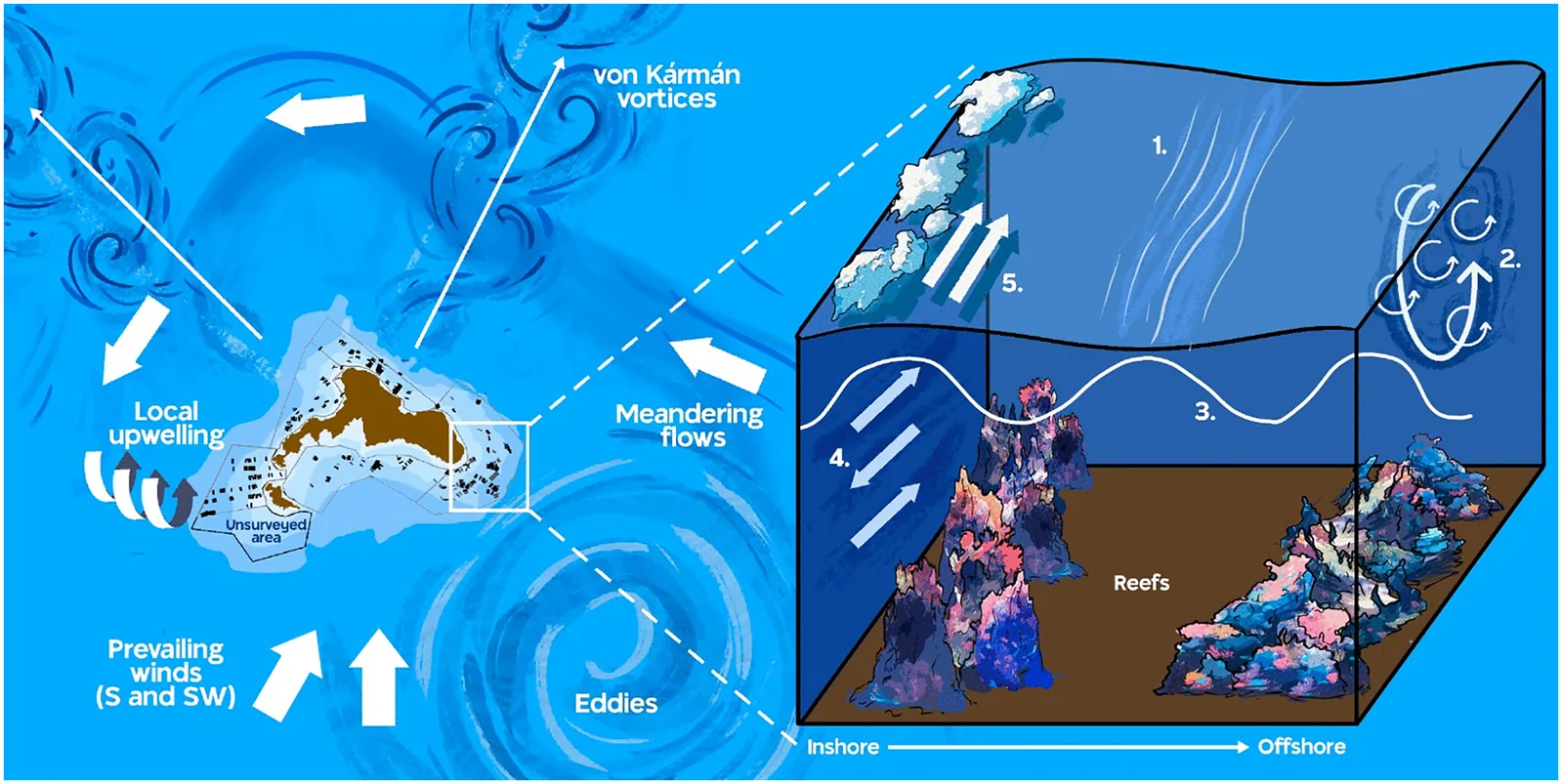
Schematic representation of reef distribution and physical forcing operating on the insular shelf. The left panel indicates the distribution of reefs along with oceanographic processes (sub– and mesoscale eddies, meander flow, von Kármán vortices, and local upwelling) identified around the coastal marine shelf of Robinson Crusoe and Santa Clara Islands. The right panel shows the distribution of reefs based on depth, where coastal reefs are taller and numerous, while reefs located at greater depth are longer and less numerous. Potential small–scale physical processes that could occur linked to reefs are shown in the right panels: tidal front (1), turbulence mixing (2), internal wave mixing (3), transport (4), surface winds (5). Credit: diagram by Felipe Gamonal.

### Reef–fish relationship

The study of the fish community on RC–SC Islands has mainly focused on taxonomic characterization [17,38,61], trophic ecology [40], and association with the shallow subtidal environment (<30 m depth; [39]). This study demonstrated that across a bathymetric range up to ~150 m on the insular shelf, reefs strongly influenced the presence of demersal fish assemblages (~0.9 probability), unlike reef–less (or flat) seafloor sites (~0.2–0.3 probability). The link between demersal fish assemblages and reefs aligns with fishers’ traditional and historical knowledge, which predicts a strong spatial correlation between higher demersal finfish catch rates and the presence of temperate rocky reefs [30,32]. Therefore, this finding provides evidence to support the fishers’ local knowledge, which is key for evidence–based decision–making and for enhancing governance in eventual conservation actions and fishery management [62,63].

Detecting spatial heterogeneity while linking reefs to fish distribution in turn facilitates the design of future assessment plans. The conservation status of fish stocks would thus be assessed based on periodic (annual/biennial) hydroacoustic surveys and on biological–fisheries monitoring of the finfish fishery, which has been in place since 2012 but with limited spatial coverage [64,65]. Studies of fish population dynamics and fishery management have shown that incorporating spatial structure is important for implementing effective management actions [66–68]. In the case of the JFA system, its heterogeneous seascape suggests spatial management actions for the exploited resources.

Like in the RC–SC Islands, the Hawaiian Islands in the North Pacific Ocean basin also feature seafloor high relief [8], which control the density and richness of herbivorous, benthic fish–carnivores and planktivores [69]. However, this study also reports fish outside the reefs on the RC–SC Island shelf (Figs 6 and 7). Identifying the fish distribution on both reef and outside reef habitats has provided clear evidence of the use of these habitats by fish species within the same survey area, indicating a use of the seascape beyond the reefs [70]. In zone 1 (NE insular shelf), for instance, the distance to the closest reef was the best fish aggregation distribution predictor based on a simple regression model approach (see Table 6). Similar results were found in the Coral Triangle region of Australia, where typical coral fish were reported in other areas without reefs, possibly due to the search for new habitats driven by ontogenetic movements of the species, daily feeding, and fishing activity [70,71].

### Spatial patterns

The sheltered region to the NE of the RC–SC Islands (zone 1) did not follow the same pattern as the other zones. Although the presence of reefs was also significant, the presence of fish was better explained by the distance to such bathymetric features. Even though the hydroacoustic survey designs implemented were not initially configured to address spatial and temporal aspects in zones 1 and 2, the depth–related pattern of fish presence/absence was similar across both seasons for the different zones, but zone 1 displayed larger variability in the probability of observing aggregations of fish at the reef sites, driven by a strong positive interaction between reef presence and the 2016 season (S4 Table). High seafloor heterogeneity in zone 1 also predicted high reef density, strengthening the spatial link not only between fish and individual reefs but also suggesting a larger spatial influence around the reefs [60,72].

Similarly, the NW shelf (zone 5) exhibited a particularly distinct spatial behavior. While reefs best predicted fish presence, detection probability was independent of depth, remaining remarkably constant across the bathymetric range over reefs (~0.65 probability) and in reef–less areas (~0.15 probability; Fig 7F). The high density of tall, narrow reefs in this area provides high seafloor complexity, maintaining stable habitat availability that uncouples fish presence from the dome–shaped bathymetric pattern. Although seafloor depth is typically considered a primary structuring factor for demersal communities, localized physical complexity across multiple spatial scales combined with key biophysical and oceanographic drivers can override this gradient, relegating depth to a secondary role in shaping fish spatial distribution [73,74].

The scale of oceanographic features impacting reefs has a broad range. For instance, the spatial influence beyond reefs on fish distribution could be based on meso– and sub meso–scale eddies and their coupled biogeochemical processes, which could modulate specific habitat conditions resulting in an attractor for the fish communities [72–76]. Like in seamount habitats, vorticity and Taylor columns [7,77] may influence circulation patterns that create patchiness in reef ecosystems [78]. On the other hand, tidal currents, turbulence, and internal waves affect the physical dynamics at small scales, promoting mixing around reefs [79,80]. These features contribute to the formation of a highly variable and dynamic zone (Fig 10), which also stands out for having the highest fish density around the RC–SC Islands [15]. While disentangling the association between fish aggregations and reefs requires a deeper understanding of the interplay between environmental and ecological processes, conservation and management plans should start considering the relevance of spatial heterogeneity for finfish communities at a broader spatial scale. Consequently, the northern sector of the RC–SC Islands (zone 1 and 5) could, therefore, require a larger sampling and monitoring effort than other zones, not focused only on reefs but on larger areas around them as well.

### Modulating factors

Intrinsic characteristics of the reefs, such as height, length, and bathymetric location, were key drivers modulating the backscatter of demersal fish aggregations, though their influence exhibited spatial heterogeneity around the RC–SC Islands. Research indicates that coastal marine shelves adjacent to taller, longer, and shallower reefs exhibit higher fish acoustic relative density. This is attributed to positive correlations between reef height and length with fish density, while average reef depth demonstrates an inverse relationship (Fig 9). Among the above characteristics, both reef length and height were the most important predictors in explaining changes in fish acoustic relative density across zones, similar to what has been observed in other studies showing that metrics related to seafloor structures are strongly associated with fish assemblage structure, including richness [81,82], diversity [58], and abundance [83–85].

Modelling fish presence and acoustic density with respect to covariates, such as depth, indicated a higher probability of detecting demersal fish aggregations and higher densities at shallower reefs than at deeper reefs (Fig 7 and 9A). However, while depth did not constrain fish presence in zone 5, was as the primary predictor of fish relative density (explaining 28% of variance), decreasing sharply toward deeper reefs. This highlights a clear decoupling between fish presence and density on the NW shelf, while the rugged seascape sustains demersal fish presence across all depths, larger aggregations preferentially concentrate on shallower and taller reef structures. The high heterogeneity that characterizes shallower areas offers more protection, which could reduce predation pressure [86,87], an interaction that contributes largely to the natural mortality of reef fish communities [88]. In coastal and shallow areas, mainly in the northeastern region (<26 m depth in zone 1), important catches of trevally/little–trevally are obtained with handline from dense pelagic aggregations (outside of this research focus) or from the upper layer of multispecies aggregations [15,16]. Whereas JF morwong catch, a central species for human consumption and lobster fishery operation, occurs on fishing grounds that match reef sites located on average at ~100 m depth contours around islands [16], where they are caught with vertical longlines laid out in the lower layer of fish aggregations linked to the seafloor. The JF morwong catch in reef sites also shows an increase in mean size as a function of depth [15]. Hence, it is expected that frequent and dense fish aggregations of JF morwong juveniles/recruits prefer habitats with substantial shelter provided by numerous and rugged reefs located in shallow areas, where the main JF morwong catches would not be developed [30].

Oceanographic processes also play a key role in structuring the environment around the seascape at different scales [89,90]. Processes such as von Kármán vortices, local upwelling, island mass effect [19,21], meanders, surface [91], and subsurface eddies [20,92,93] have impacts on island productivity (Fig 10; [23]). However, the interactions of these physical processes with the different structures on the insular shelf (from discrete reefs to larger groups of them) require additional knowledge to fully assess their roles in the ecological connection between fish and reefs. Thus, while productivity and food availability significantly influence fish distributions, their effects interact with reef structure and oceanographic dynamics, highlighting that multiple interacting factors collectively shape fish presence and aggregations across the insular shelf.

### Toward management and conservation

The use of hydroacoustic methods has enabled the acquisition of comprehensive, continuous, high–precision spatial and temporal information on the entire demersal finfish community of the insular shelf without compromising the fragile ecosystem. These methods have been key to understanding the structure and heterogeneity of the insular marine shelf landscape at the spatial scale (Fig 10). It also highlighted the ecological value of different reef systems and established the foundations for subsequent study designs, using a combination of hydroacoustic and optical tools [25,37]. High–resolution mapping of reef characteristics could enhance an area–based design, a key to conservation and fishing management plans. More accurate surveys on rocky reefs, however, require more sophisticated methodologies such as multibeam echosounders to scan the seabed [37]. Using stereo cameras coupled with hydroacoustic equipment may result in quantitative estimation of species proportions and the size structure of demersal fish aggregations [25,94], ideally by depth strata. Although only 38 kHz was used in this survey, future work integrating multi–frequency analyses and target–strength estimations could improve species–level discrimination in reef habitats [37,44,95,96]. These methods would provide more robust indicators of abundance and biomass for key endemic fishes [97–99], such as the JF morwong, thereby supporting both conservation and fishery management. This approach will eventually enable monitoring fish abundance across their entire bathymetric range and optimize sampling effort in areas with a high probability of detecting them (above and/or near the reefs; Fig 10).

A cost–effective surveying approach targeting reefs is of particular interest in the remote JFA, as finfish species are highly vulnerable and of high subsistence and economic value [32,33]. This work linked rocky reefs’ spatial heterogeneity to fish distribution, establishing the seascape fundamentals for subsequent survey designs and conservation plans within JFA Marine Protected Area. To enhance the prospects of implementing an ecosystem–based conservation plan, gaining a more thorough understanding of the sources and dynamics of physical, chemical, and biological processes across various spatio–temporal scales (see Fig 10; [5,23]). Furthermore, evaluating their influence on conservation efforts [6] and considering the socio–economic aspects of the Juan Fernández community [100–102] can contribute to a more comprehensive outlook and better management.

## Supporting information

S1 Table Model selection for the presence–absence of fish aggregation. Results of regression models (GAMLSS) evaluated via stepwise inclusion of zone as a fixed factor and its interactions with primary predictors. Covariates: *pr*_*l*_ represents reef presence, *z*_*l*_ represents seafloor depth, *dist*_*l*_ represents distance to the reef, and *s*(·) indicates a P–spline smooth function. Model descriptors: *df* corresponds to degrees of freedom, *AIC* represents Akaike information criterion, and *ΔAIC* to the difference relative to the best model. Model performance was evaluated using AIC, with lower values indicating a better fit. Bold values denote the best–fitting model.
(DOCX)

S2 Table Parameter estimates for the best–fitting model (*M5*). Model coefficient estimates (β), standard errors (Std. Error), *t*–values, and *p*–values for the presence–absence of demersal fish aggregations. Zone 2 was the baseline reference level for categorical comparisons. Statistical significance was set at *p* < 0.05.
(DOCX)

S3 Table Spatial and temporal model selection for presence–absence of demersal fish aggregations. Results of regression models (GAMLSS) evaluating spatial and temporal consistency across zones 1–2 and survey season 2015–2016. Models evaluate an additive season effect as baseline model (*ST*_*0*_) against a triple interaction among the linear primary predictors, zones, and season (*ST*_*1*_). Covariates: *pr*_*l*_ represents reef presence, *z*_*l*_ represents seafloor depth, *dist*_*l*_ represents distance to the reef, season represents survey year, and *s*(·) indicates a P–spline smooth function. Model descriptors: *df* corresponds to degrees of freedom, *AIC* represents Akaike information criterion, and *ΔAIC* to the difference relative to the best model. Model performance was evaluated using AIC, with lower values indicating a better fit. Bold values denote the best–fitting model.
(DOCX)

S4 Table Parameter estimates for the best–fitting spatial and temporal model (*ST*_*1*_). Model coefficient estimates (β), standard errors (Std. Error), *t*–values, and *p*–values for the presence–absence of demersal fish aggregations. Zone 2 and sampling season 2015 were the baseline reference levels for categorical comparisons. Statistical significance was set at *p* < 0.05.
(DOCX)

S5 Table Model selection for backscatter of fish aggregation. Results of regression models fitted to the continuous response variable (fish aggregation backscatter) via stepwise inclusion of zone as a fixed factor and its interactions with reef attribute predictors. Covariates: *Zr*_*h*_ represents mean reef depth, *Lr*_*h*_ to reef length, and *Hr*_*h*_ to average reef height, and *s*(·) indicates a P–spline smooth function. Model descriptors: *df* corresponds to degrees of freedom, *AIC* represents Akaike information criterion, and *ΔAIC* to the difference relative to the best model. Model performance was evaluated using AIC, with lower values indicating a better fit. Bold values denote the best–fitting model.
(DOCX)

S6 Table Parameter estimates for the best–fitting model (*M10*). Model coefficient estimates (β), standard errors (Std. Error), *t*–values, and *p*–values for the backscatter of demersal fish aggregations. The intercept represents the expected global baseline backscatter when reef covariates are zero. Statistical significance was set at *p* < 0.05.
(DOCX)

S1 Fig Assessing reef coverage, length, and density across zones and depths. Proportion of reef covered area (reef area/ sampled area), reef median length (km), and reef density [(number of reefs/ km^2^) × 1000^−1^] by (A) zone and (B) depth strata.
(TIF)
