# Supplementary material for: Understanding spatial heterogeneity of rocky reef demersal fish assemblages: Links to conservation plans in the Juan Fernández Archipelago Marine Protected Area in the Southeastern Pacific
Source: PLoS One. 2026 Oct 9;21(10):e0358459. doi: 10.1371/journal.pone.0358459 (PMC13652157; doi:10.1371/journal.pone.0358459)
Supplement: Supplementary file 1 [file pone.0358459.s001.docx]

| Model | Covariate | df | Global deviance | AIC | ΔAIC |
| --- | --- | --- | --- | --- | --- |
| M0 | ~pr_l_ + *s*( z_l_) + dist_l_ | 12 | 6563 | 6588 | 527 |
| M1 | ~pr_l_ + *s*( z_l_) + dist_l_ + zone | 16 | 6339 | 6370 | 309 |
| M2 | ~pr_l_ × zone + *s*( z_l_) + dist_l_ | 20 | 6273 | 6312 | 251 |
| M3 | ~pr_l_ × zone + *s*( z_l_) × zone + dist_l_ | 35 | 6147 | 6218 | 157 |
| M4 | ~pr_l_ × zone *+ s*( z_l_) × zone + dist_l_ × zone | 35 | 5992 | 6063 | 2 |
| **M5** | **Saturated model (M4) except z_l_ in zone 5** | **34** | **5992** | **6061** | **0** |
