# Supplementary material for: Understanding spatial heterogeneity of rocky reef demersal fish assemblages: Links to conservation plans in the Juan Fernández Archipelago Marine Protected Area in the Southeastern Pacific
Source: PLoS One. 2026 Oct 9;21(10):e0358459. doi: 10.1371/journal.pone.0358459 (PMC13652157; doi:10.1371/journal.pone.0358459)
Supplement: Supplementary file 2 [file pone.0358459.s002.docx]

| **Covariate** | **Estimate (β)** | **Std. Error** | **t-value** | **p-value** |
| --- | --- | --- | --- | --- |
| intercept (zone 2) | 0.481 | 0.240 | 2.005 | 0.045 |
| zone 1 | -0.649 | 0.309 | -2.099 | 0.036 |
| zone 3 | -0.646 | 0.494 | -1.309 | 0.191 |
| zone 4 | -2.130 | 0.464 | -4.592 | <0.001 |
| zone 5 | -2.146 | 0.297 | -7.226 | <0.001 |
| reef presence (pr_l_) | 3.463 | 0.259 | 13.355 | <0.001 |
| pr_l_ × zone 1 | -1.873 | 0.316 | -5.927 | <0.001 |
| pr_l_ × zone 3 | 0.522 | 0.439 | 1.189 | 0.235 |
| pr_l_ × zone 4 | -0.900 | 0.394 | -2.285 | 0.022 |
| pr_l_ × zone 5 | -1.157 | 0.364 | -3.183 | 0.002 |
| seafloor depth (*s*(z_l_) × zone 1) | 0.004 | 0.002 | 2.238 | 0.025 |
| *s*(z_l_) × zone 2 | -0.006 | 0.002 | -3.073 | 0.002 |
| *s*(z_l_) × zone 3 | -0.027 | 0.004 | -6.849 | <0.001 |
| *s*(z_l_) × zone 4 | -0.002 | 0.003 | -0.477 | 0.633 |
| distance to reef (dist_l_) | -0.002 | 0.000 | -13.742 | <0.001 |
| dist_l_ × zone 1 | -0.001 | 0.000 | -2.752 | 0.006 |
| dist_l_ × zone 3 | 0.003 | 0.000 | 11.412 | <0.001 |
| dist_l_ × zone 4 | 0.002 | 0.001 | 2.888 | 0.004 |
| dist_l_ × zone 5 | 0.002 | 0.000 | 8.741 | <0.001 |
