# Supplementary material for: Understanding spatial heterogeneity of rocky reef demersal fish assemblages: Links to conservation plans in the Juan Fernández Archipelago Marine Protected Area in the Southeastern Pacific
Source: PLoS One. 2026 Oct 9;21(10):e0358459. doi: 10.1371/journal.pone.0358459 (PMC13652157; doi:10.1371/journal.pone.0358459)
Supplement: Supplementary file 3 [file pone.0358459.s003.docx]

| Model | Covariate | df | Global deviance | AIC | ΔAIC |
| --- | --- | --- | --- | --- | --- |
| ST_0_ | ~pr_l_ × zone *+ s*( z_l_) × zone + dist_l_ × zone + season | 17 | 4015 | 4049 | 30 |
| **ST_1_** | **~pr_l_ × zone × season *+ s*( z_l_) × zone + dist_l_ × zone × season** | **21** | **3976** | **4019** | **0** |
