# Supplementary material for: Understanding spatial heterogeneity of rocky reef demersal fish assemblages: Links to conservation plans in the Juan Fernández Archipelago Marine Protected Area in the Southeastern Pacific
Source: PLoS One. 2026 Oct 9;21(10):e0358459. doi: 10.1371/journal.pone.0358459 (PMC13652157; doi:10.1371/journal.pone.0358459)
Supplement: Supplementary file 4 [file pone.0358459.s004.docx]

| **Category** | **Covariate** | **Estimate (β)** | **Std. Error** | **t-value** | **p-value** |
| --- | --- | --- | --- | --- | --- |
| Base | intercept (zone 2, 2015) | 0.420 | 0.282 | 1.493 | 0.135 |
| Main fixed effects | zone 1 | -0.337 | 0.351 | -0.962 | 0.336 |
|  | reef presence (pr_l_) | 2.536 | 0.351 | 7.219 | <0.001 |
|  | distance to reef (dist_l_) | -0.003 | 0.000 | -7.329 | <0.001 |
|  | season 2016 | -0.133 | 0.201 | -0.660 | 0.510 |
| Spatial interactions | pr_l_ × zone 1 | -1.474 | 0.406 | -3.632 | <0.001 |
|  | s(z_l_) × zone 1 | 0.003 | 0.002 | 1.922 | 0.055 |
|  | s(z_l_) × zone 2 | -0.005 | 0.002 | -2.472 | 0.014 |
|  | dist_l_ × zone 1 | -0.001 | 0.001 | -2.161 | 0.031 |
| Temporal interactions | pr_l_ × season 2016 | 1.695 | 0.554 | 3.059 | 0.002 |
|  | dist_l_ × season 2016 | 0.001 | 0.000 | 2.015 | 0.044 |
|  | zone 1 × season 2016 | 0.158 | 0.299 | 0.528 | 0.598 |
| Three-way interactions | pr_l_ × zone 1 × season 2016 | 0.263 | 0.800 | 0.328 | 0.743 |
|  | distl × zone 1 × season 2016 | 0.001 | 0.001 | 1.520 | 0.129 |
