# Supplementary material for: Understanding spatial heterogeneity of rocky reef demersal fish assemblages: Links to conservation plans in the Juan Fernández Archipelago Marine Protected Area in the Southeastern Pacific
Source: PLoS One. 2026 Oct 9;21(10):e0358459. doi: 10.1371/journal.pone.0358459 (PMC13652157; doi:10.1371/journal.pone.0358459)
Supplement: Supplementary file 5 [file pone.0358459.s005.docx]

| Model | Covariate | df | Global deviance | AIC | ΔAIC |
| --- | --- | --- | --- | --- | --- |
| M6 | ~*s*(Zr_h_) + *s*(Lr_h_) + *s*(Hr_h_) | 8 | 4969 | 4986 | 28 |
| M7 | ~*s*(Zr_h_) + *s*(Lr_h_) + *s*(Hr_h_) + zone | 12 | 4947 | 4971 | 14 |
| M8 | ~*s*(Zr_h_) × zone + *s*(Lr_h_) + *s*(Hr_h_) | 15 | 4930 | 4960 | 2 |
| M9 | ~*s*(Zr_h_) × zone + *s*(Lr_h_) × zone + *s*(Hr_h_) | 23 | 4917 | 4963 | 6 |
| **M10** | **~*s*(Zr_h_) × zone + *s*(Lr_h_) × zone + *s*(Hr_h_) × zone** | **29** | **4900** | **4958** | **0** |
| M11 | ~*s*(Zr_h_) × zone + *s*(Lr_h_) × zone + *s*(Hr_h_) × zone | 29 | 4900 | 4958 | 0 |
