# Supplementary material for: Understanding spatial heterogeneity of rocky reef demersal fish assemblages: Links to conservation plans in the Juan Fernández Archipelago Marine Protected Area in the Southeastern Pacific
Source: PLoS One. 2026 Oct 9;21(10):e0358459. doi: 10.1371/journal.pone.0358459 (PMC13652157; doi:10.1371/journal.pone.0358459)
Supplement: Supplementary file 6 [file pone.0358459.s006.docx]

| **Covariate** | **Estimate (β)** | **Std. Error** | **t-value** | **p-value** |
| --- | --- | --- | --- | --- |
| Intercept | 7.661 | 0.220 | 34.758 | <0.001 |
| *s*(Zrh) × zone 1 | -0.011 | 0.002 | -4.923 | <0.001 |
| *s*(Zrh) × zone 2 | -0.009 | 0.002 | -4.578 | <0.001 |
| *s*(Zrh) × zone 3 | -0.023 | 0.004 | -5.751 | <0.001 |
| *s*(Zrh) × zone 4 | -0.025 | 0.003 | -7.574 | <0.001 |
| *s*(Zrh) × zone 5 | -0.026 | 0.004 | -7.266 | <0.001 |
| *s*(Lrh) × zone 1 | 0.010 | 0.001 | 10.069 | <0.001 |
| *s*(Lrh) × zone 2 | 0.006 | 0.001 | 8.643 | <0.001 |
| *s*(Lrh) × zone 3 | 0.003 | 0.001 | 2.212 | 0.028 |
| *s*(Lrh) × zone 4 | 0.003 | 0.001 | 2.744 | 0.007 |
| *s*(Lrh) × zone 5 | 0.002 | 0.002 | 0.824 | 0.411 |
| *s*(Hrh) × zone 1 | 0.011 | 0.018 | 0.631 | 0.529 |
| *s*(Hrh) × zone 2 | 0.085 | 0.032 | 2.628 | 0.009 |
| *s*(Hrh) × zone 3 | 0.179 | 0.074 | 2.411 | 0.017 |
| *s*(Hrh) × zone 4 | 0.369 | 0.069 | 5.388 | <0.001 |
| *s*(Hrh) × zone 5 | 0.214 | 0.037 | 5.862 | <0.001 |
